# Paper-based facile capacitive touch arrays for wireless mouse cursor control pad

**DOI:** 10.1016/j.heliyon.2023.e19447

**Published:** 2023-08-26

**Authors:** Myda Arif, Muhammad Hamza Zulfiqar, Muhammad Atif Khan, Muhammad Zubair, Muhammad Qasim Mehmood, Yehia Massoud

**Affiliations:** a MicroNano Lab, Department of Electrical Engineering, Information Technology University (ITU) of the Punjab, Ferozepur Road, Lahore, 54600, Pakistan; b Department of Biomedical Engineering, Narowal Campus, University of Engineering and Technology (UET), Lahore, 54890, Pakistan; c Innovative Technologies Laboratories (ITL), King Abdullah University of Science and Technology (KAUST), Saudi Arabia

**Keywords:** Wireless mouse, Touch arrays, Touchpad, Capacitive sensor, Paper-based

## Abstract

Wireless devices have become extremely inexpensive and popular in recent years. The two most significant advantages of wireless devices over wired ones are convenience and flexibility. Considering this, a wireless mouse pad prototype for access has been developed in this study. A capacitive sensors-based mouse pad with basic operations and conventional features has been developed using sensing arrays on paper. A facile, do-it-yourself fabrication process was utilized to develop a cost-effective, thin, wearable, and cleanroom-free wireless mouse cursor control (MCC) pad. The ablation process was used to cut the traces of conductive tape and paste them onto the paper to develop the MCC pad. The pad was connected with Espressif Systems (ESP)32 to wirelessly control the cursor of mobile and laptop. The capacitive touch sensor array-based pad is easy to reproduce and recycle. This pad can contribute to future advancements in thin human-machine interfaces, soft robotics, and medical and healthcare applications.

## 1 Introduction

Since the current wired mouse approach is insufficiently effective for flexibility and wire autonomy. The users then adopt the wireless mouse concept [1], which has numerous distinctive advantages. With the advancement of technology in the fields of wearable technology [2] and ubiquitous devices, these devices are becoming more modest in their use of Bluetooth or wireless technologies [3]. High performance, versatility, lightweight, and mass production are only a few of the criteria for flexible sensors [4] that have been raised by the rapidly evolving visualization technology as well as the enormous different devices and Internet of Things markets [5]. Flexible electronics and wearable electronic devices have demonstrated a considerable application potential for flexible sensing technology [6], which has also received extensive research. Flexible sensors with resistive, capacitive, inductive, triboelectric, and other sensing techniques were used to measure mechanical amounts [[6], [7], [8], [9], [10], [11], [12]]. Due to its low price, the lightweight, easily accessible cellulose-based paper has gained popularity recently. These materials are frequently used for commercial purposes, information storage and packaging, which include strain sensors [9], solar cells [10], supercapacitors, photovoltaic circuits, reusable HMI sensors, gas sensors, stretchable electronics [12], displays, heart rate sensors, humidity sensors, multi-responsive flexible sensors and touch sensors are examples of developments in foldable and flexible electronics based on a paper substrate [[13], [14], [15], [16], [17], [18], [19], [20], [21], [22], [23], [24], [25], [26], [27]].

Moreover, the importance of wireless technology is increasing. One of the biggest benefits of wireless devices [28] over wired ones is convenience, followed by versatility. When it comes to flexibility and the disruptions brought on by a connected connection, the application of a wired mouse is insufficiently effective. This is the biggest factor behind the present spike in users as they move to a world with effective wireless options. These wireless devices, such as wireless mouse [[29], [30], [31]], headphones, and keyboards [32], have the major benefit of clearing up the mess of wires surrounding the workspace. The convenience of work and refinement of the finished product may therefore improve. To create touch sensors, a wide range of technologies can be used. Most depend on capacitive and resistive technologies, like many capacitive touch pads [33] and tactile sensors [34,35]. The innovation disclosed herein relates, in various embodiments, to the area of touchscreens and touchpads [36] generally, and in particular, devices, components, and methods for measurement or detection that integrate mutual capacity and radio frequency are particularly useful in touch-sensitive or touchscreen systems for portable electronic devices [37]. Embodiments of the invention disclosed herein include those for a single-finger, single-use movable cursor in portable or portable devices [38], such as smartphones, iPods, computer screens, video game consoles, laptops, iPads, etc. This capacitive touchpad does not require any pressure or force to operate. Instead, lightly place your finger on the touchpad. Another benefit of this wireless capacitive Touchpad is that they are a system that can support multiple screens touches simultaneously if modified. When using this capacitive touchpad, which can detect multiple points of contact, such as when zooming with two fingers, you will need special modifications in addition to the pad itself.

The objective is to design a user-friendly touch screen for a mouse system. Capacitive touchscreens use the variation in capacitance caused by the finger to identify the touch's area. Comparatively, to resistive methods [39], which necessitate considerable pressure actually to make contact between two conducting layers, capacitive technologies may sense a change in capacitance simply by placing a small touch on the screen. As a result, it enables speedy and smooth movement, excellent durability and superior optical performance. Additionally, unlike resistive systems, which necessitate at least one flexible layer, layers can be constructed of any material, including paper, plastic, or glass. Here, the proposed idea is to use a capacitive sensor to operate the cursor. The sensors were made from inexpensive, non-toxic, and e-waste-free materials, including paper and copper tape. With the help of the ESP32 microcontroller, which behaves like a Bluetooth mouse, you can control its actions. For instance, move the mouse upward, downward, left, right, and click, etc. One may easily operate the mouse cursor with the help of a finger touch screen without worrying about the cable being knotted. The prototype in this paper discusses the design of a mouse cursor with wireless control and capacitive touch sensors after this operation. The advantage of the device is that it can be configured to function as a mouse because it is microcontroller-based. A capacitive touch sensor has been considered an alternative to an optical mouse's [39] reflective surface. In addition, an ESP32 BLE library has been used to implement the wireless feature [40].

## 2 Fabrication and experimental setup

### 2.1 Working principle

**Fig. 1:**
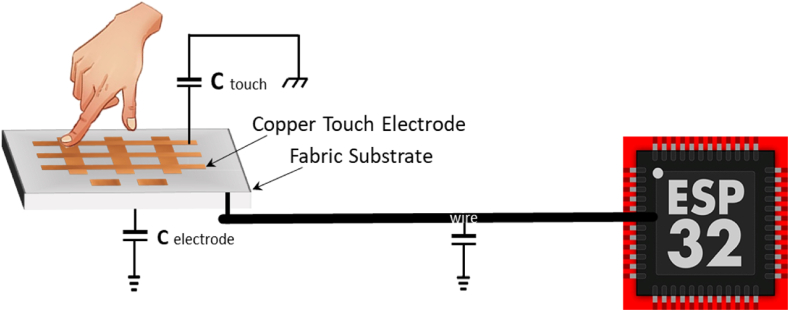
Side view representation of flexible hybrid design capacitance sensor.

Capacitive sensing technology [41] works by monitoring the change in capacitance (a) system's ability to store an electric charge) within its extended field because of the presence of a conductive object. Typically, such an object is a human finger, but it might also be any conductive object with a different dielectric than air. A single capacitive electrode connected to an ESP32 microcontroller is shown in Fig. 1. A parallel plate capacitor is created between the electrode and the finger when a user contacts the electrode. This implies that the capacitance of the touch channel will change if a user touches the electrode. Using a microcontroller, we can then interpret this change in capacitance to indicate that there has been contacted with the electrode surface. The total capacitance is given as

[1] $$C_{total}=C_{internal}+C_{wire}+C_{electrode}+C_{touch}$$

where C total is the entire capacitance of the touch sensor, C internal is its intrinsic parasitic capacitance, C wire is its parasitic capacitance, C electrode is its parasitic capacitance, and C touch is the capacitance that the finger introduces when it touches the touch sensor. C internal, C wire, and C electrode are fixed parasitic capacitances Cp as given in equation [1]. A parallel plate capacitor is created because of the conductivity of the finger and the body's mass functioning as an earth ground when a finger is put on a touch sensor, changing the capacitance of C touch in accordance with equation [1].

**Fig. 2:**
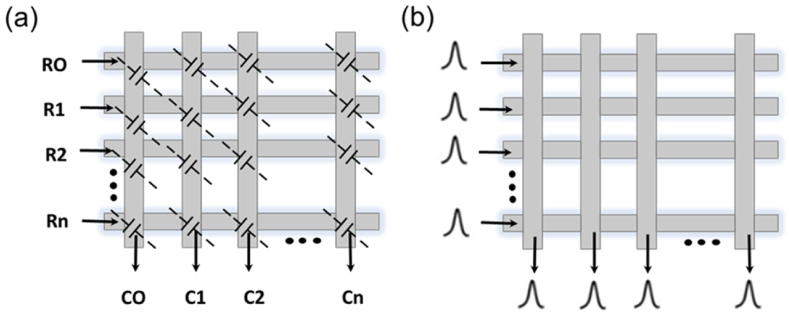
Detailed Illustration of electrode components at the intersections of rows and columns in a two-dimensional sensor. (a) Self Capacitance. (b) Mutual Capacitance.

The development of a capacitive sensor includes six electrodes in the grid and two in the clicks covered by a thin, insulating surface. This wireless capacitive wearable mouse uses 8 electrodes. In general, projected-capacitive touchscreen panels employ two patterned conductive layers isolated and crossed to form a matrix. Horizontal and vertical patterns correspond to touch event position information [42]. While self-capacitance detects capacitance between layers and the ground, as shown in Fig. 2 (a), mutual capacitance detects capacitance in the overlapped areas of horizontal and vertical patterns, as shown in Fig. 2 (b). As a result, the finger touch increases self-capacitance due to the additional parasitic capacitor in parallel while decreasing mutual capacitance due to electric field loss caused by the finger being placed between two electrodes. The capacitance of vertical and horizontal electrodes placed over the ground is used to estimate the x-axis and y-axis coordinates by self-capacitance. As a result, multiple touches may result in ghost touches. However, because mutual capacitance measures the overlap capacitance between vertical and horizontal conductive patterns separately, it can support multi-touch functions with no limit on the number of fingers. Furthermore R0, R1, R2 and CO, C1, C2 are basically ×0, ×1, ×2 and y0, y1, y2. By using four different combinations of two x-axis data and two y-axis data, the mutual capacitance, for instance, can be used to determine that there are two touches at locations (×1, y1) and (×2, y2), respectively. It can then provide two correct locations, (×1, y1) and (×2, y2) [43,[44], [45]].

### 2.2 Fabrication process

**Fig. 3:**
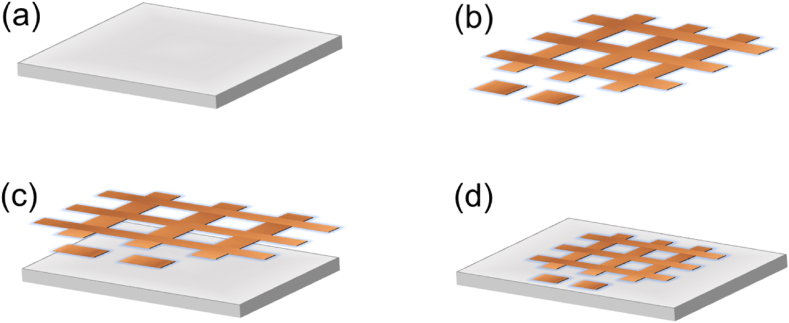
Fabrication of copper tape-based capacitive touch arrays for MSS pad. (a) The lower side of A4 paper. (b) Copper-tape arrays. (c) The upper side of A4 paper with arrays. (d) Fabricated sensor.

The sensor electrodes are made using an innovative technology that uses readily available, eco-friendly materials. The copper-based, parallel-plate capacitive touch sensor is shown in Fig. 3. It is inexpensive, reliable, recyclable, and easily replaced. The grid for the capacitive touch sensor was made of copper. Each copper strip has dimensions of 8 cm × 1 cm and 2 cm × 2 cm for squares. The layout was created using standard A4 paper. First, the copper tape is cut using a paper cutter into strips and squares corresponding to the above-mentioned measurements. After being cut to fit the measurements, these six strips and two squares of copper tape were interconnected in the form of a grid by ensuring a consistent distance between each cell. Then that 0.15 mm-thick copper tape grid is placed on a paper substrate. The detailed fabrication process is depicted in Fig. 3. In Fig. 3(a), we can see the lower side of an A4 paper, which serves as the core substrate. Fig. 3 (b) emphasises the complicated arrangement of copper-tape arrays that compose the capacitive touch sensor mechanism. Moving on to Fig. 3 (c), we see the upper side of the A4 paper with the integrated arrays, demonstrating their location. Finally, Fig. 3(d) shows the finished product - the fully constructed sensor with its copper-tape array layout. Also, this sensor is inexpensive, easy to build, and requires no special fabrication techniques.

### 2.3 Experimental setup

**Fig. 4:**
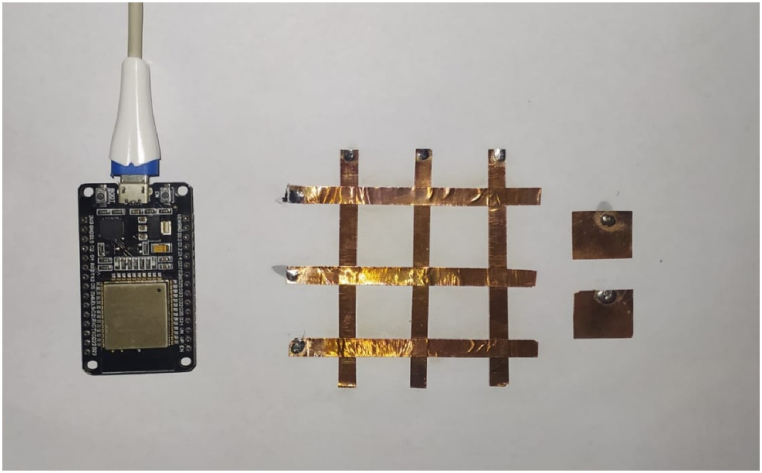
Experimental setup of capacitive touch Based Mouse Cursor Control (MCC) using ESP32.

The ESP32 microcontroller is utilized, which supports 10 capacitive-sensing GPIOs that can detect changes brought on by a finger or other object touching or coming close to the GPIOs. Jumper wires are soldered to the edges of each copper strips and squares and inserted into the ESP32. The microcontroller reports the charge transfer pulse value as the capacitor's number of charging and discharging cycles that happened during the chosen measurement time interval. A serial monitor is used to monitor the sensor's response. With the built-in Bluetooth and WIFI radio communication capabilities of microcontrollers, wireless control functionality is achieved with minimal setup, as shown in Fig. 4. The capacitive sensor array is our input. The sensor array is made up of eight copper-tape mesh sensors. The user's input is received by this array, which then delivers it to the microcontrollers, which turn it into mouse movement data packets and send them via Bluetooth to any device (desktops, laptops, and smartphones). These signals will further translate all of the events and coordinates into actions. For example, the mouse cursor is supposed to move when the touch sensor pad is touched at one coordinate and moved to another coordinate. Additionally, it determines the cursor's direction, left and right clicks, and movement distance.

## 3 Results and discussion

**Fig. 5:**
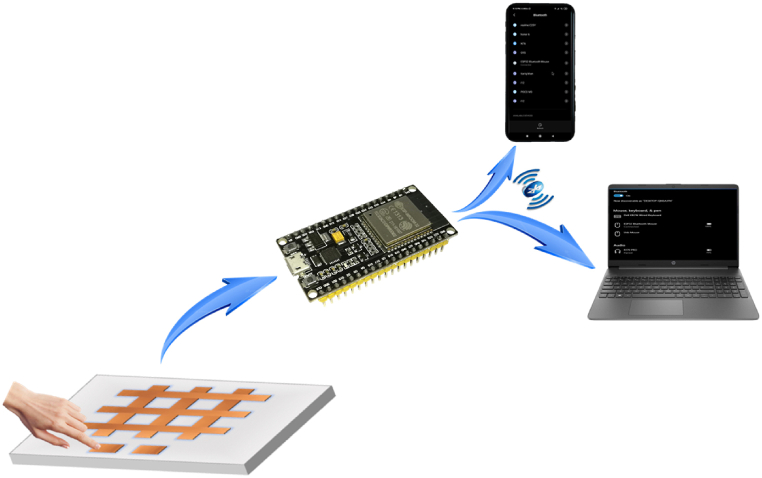
Implementation of the capacitive touchpad.

The majority of homes use capacitive screens. Capacitive sensors are capable of detecting anything that has conductive or dielectric properties. It monitors the variations in a sensor's capacitance when it is touched. Our input comes from the capacitive sensor array. This array captures the user's input and passes it on to the microcontrollers, who transform it into digital mouse movement data and transmit it via Bluetooth to any device (desktops, laptops, and smartphones) as shown in Fig. 5. These signals will further translate all the events and coordinates into actions. When the capacitive touchpad is touched at one coordinate and transferred to another, the mouse cursor should move accordingly, which controls the cursor's movements and clicks. We interact with capacitive touch screen technology the most. This type of display can be found on our tablets, computers, and smartphones, as well as on touchscreen printers, ticket machines, arcade games, ATMs, and car GPS.

**Fig. 6:**
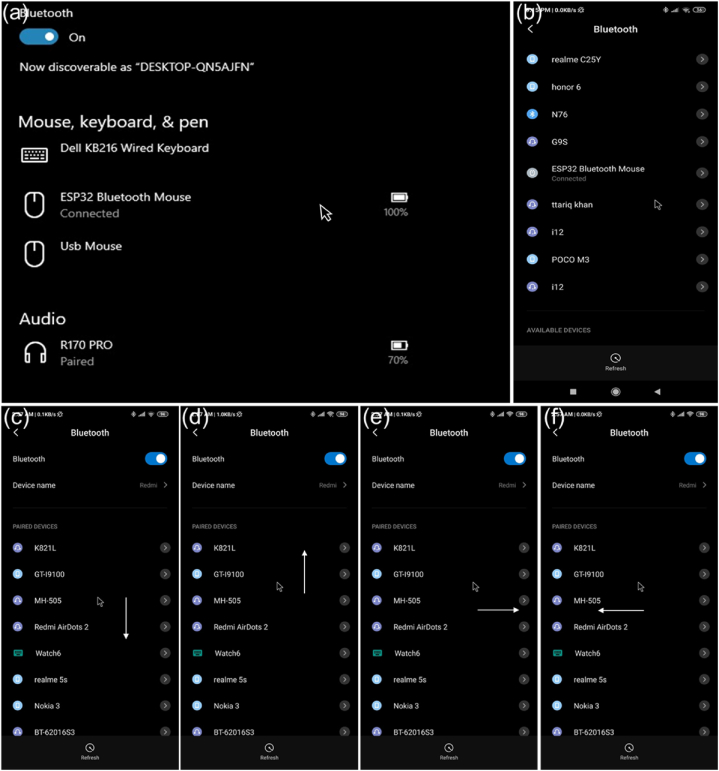
Desktop and mobile operation using wireless MCC pad. (a) MCC pad connected to a desktop. (b) MCC pad is connected to a mobile. (c) Cursor movement in the downward direction. (d) Cursor movement in the upward direction. (e) Cursor movement in the left direction. (e) Cursor movement in the right direction.

Capacitive touchscreens are found on almost all smartphones, laptops, and tablets. This means that whenever the finger makes contact with the screen or even just comes close to it, it causes a very slight electrical disturbance on its surface, which causes the phone to receive signals. Any device works reliably by operating that layout as a touch screen when connected with an ESP32 BLE mouse. Desktop and mobile connected with that microcontroller via Bluetooth show the cursor position as shown in Fig. 6 (a) and (b), respectively. The curser was moved in all possible directions as down, up, right, and left, as shown in Fig. 6 (c), (d), (e) and (f), respectively.

**Fig. 7:**
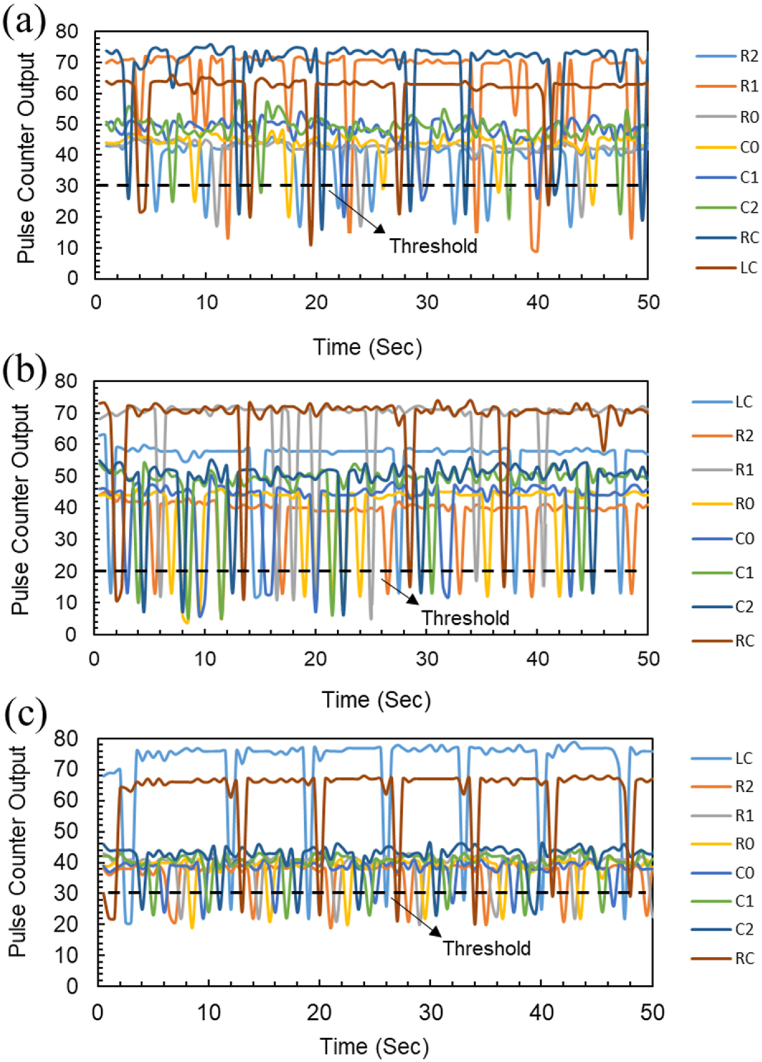
Touch sensor pulse counter output for touched and not touched state. (a) For bare finger touch response. (b) For polythene gloved touch response. (c) For nitrile gloved touch response.

Capacitive sensors measure the relative changes in a sensor's capacitance when it is touched using a switched capacitor approach. A voltage pulse transfers charges between a capacitor (Cs) and an unknown capacitance (Cx) connected in series. A high-resolution measurement system that can respond to changes in capacitance of only a few femtofarads is produced by repeatedly executing the pulse. Therefore, it is crucial to enable the voltage pulse to settle properly and transfer all charges into Cx and Cs to produce stable and consistent results. However, the RC time constants thus created will tend to slow down this settling process because Cx and Cs are typically coupled with some degree of series resistance. Therefore, it's crucial to keep in mind both the size of Cx (the sensor's capacitance) and the quantity of series resistance while building a capacitive touch system. The fabricated touch sensor showed reliable data, with no false positives found during testing. To determine the threshold, the pulse counter values of touch sensors were repeatedly tested in touch and no-touch states with bare fingers, polythene gloves, and nitrile gloves, as shown in Fig. 7 (a), (b) and (c), respectively. The fabricated capacitive arrayed MCC pad showed reliable data, of each row in x coordinate and columns in the y coordinate, with no false positives found during testing. To determine the threshold, the pulse counter values of all the points of arrays were repeatedly evaluated in touch and no-touch states with bare fingers of each row and column (R0, R1, R2 as ×0, ×1, ×2 and CO, C1, C2 as y0, y1, y2) and left and right clicks (LC and RC) as shown in Fig. S1. The response of individual points of arrays of MCC pad for polythene and nitrile gloves is shown in Figs. S2 and S3, respectively.

**Fig. 8:**
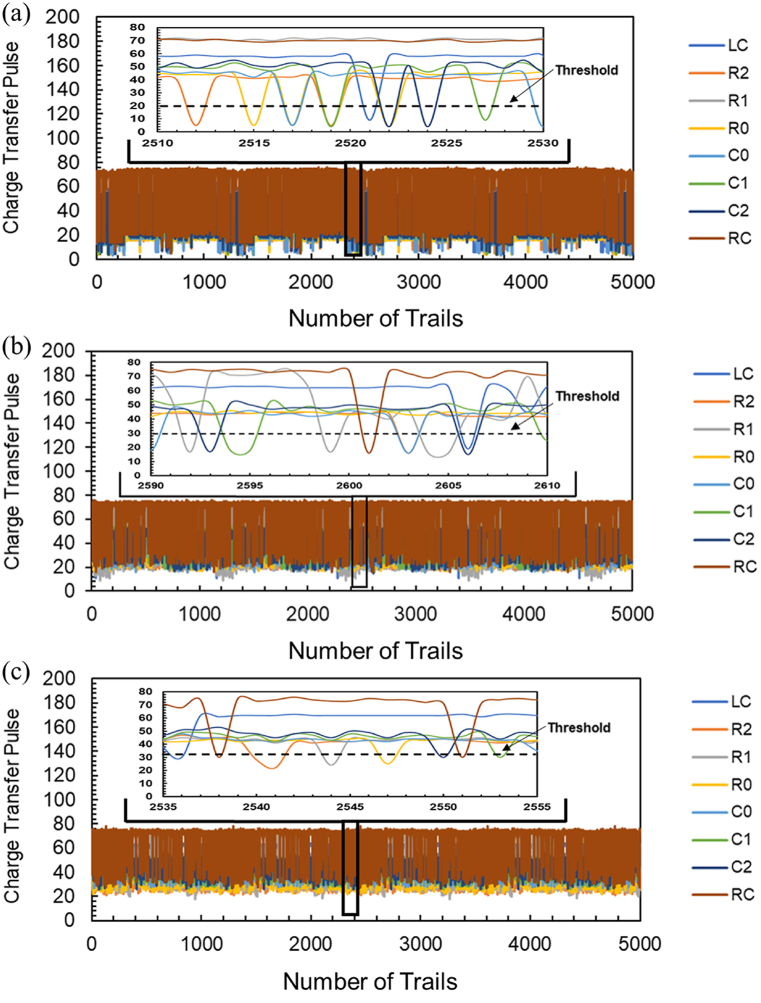
Average specific charge transfer pulse response versus cycle number (a) With a bare finger. (b) With a polyethylene glove. (c) With a nitrile glove.

In order to construct a graph and record the electrical response, we modeled and tested a paper-based touchpad by pressing each touch sensor with a bare finger multiple times and then with the polythene and nitrile gloved finger. We repeatedly touched the touchpad to prove how it worked over 1000 times, and it worked each time consistently without drifting. To check the reliability and sensitivity, the specific charge transfer pulse response versus the number of trails is shown in Fig. 8. Fig. 8 (a), (b) and (c) show the response of the MCC arrays pad for bare, polythene gloved, and nitrile gloved fingers.

A black dotted line depicts Fig. 8 (a) threshold, and it differs in (b) and (c). Measurements in Figures (b) and (c) were made with a gloved finger using the different blue dotted lines as in (a). Hence, touching a finger with a gloved hand caused a significantly smaller change in charge transfer pulse than touching a bare finger, and future advances in electronics may improve sensitivity to gloved fingers.

**Fig. 9:**
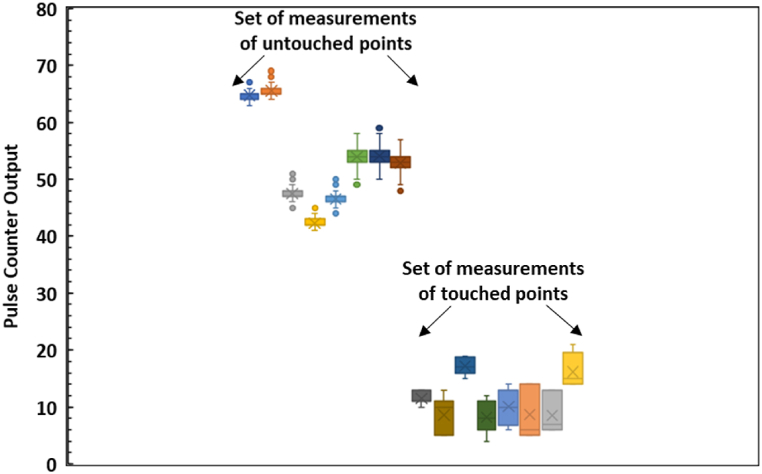
Box plot for the pulse counter output for two sets (touched and untouched) of several measurements.

Moreover, we pressed the touchpad in Fig. 2 over 2000 times to demonstrate its functionality, and it continued to function reproducibly. We also measured the touchpad's response to hundreds of presses with a bare finger, as shown in some of the measurements taken with the Arduino-based system after the touchpad had already been pressed over 1000 times. Also, the accuracy of the wireless mouse cursor control pad movement was checked by performing a series of experiments and observations of touch and not touch values, which have been demonstrated in the box plotting graph as depicted in Fig. 9.

When the capacitive MCC pad is touched at one coordinate and transferred to another, the mouse cursor should move accordingly, which controls the cursor's movements and clicks. In video S1, a nitrile glove finger is used to demonstrate the movement of the desktop cursor. First, the cursor on the desktop changes to a left click as the gloved finger clicks left on the capacitive sensor. Then it moves diagonally from top to bottom, to the right, and moves accordingly. In Video S2, the test was performed with a bare finger for the movement of the cursor on the desktop. The cursor on the desktop changes to a left click as the finger clicks left on the capacitive sensor. Then it moves upward to downward, then downward to upward, and so on. Video 3 demonstrates the cursor movement on a smartphone reliably upward to the left, then downward to the left, and so on.

Supplementary data related to this article can be found online at https://doi.org/10.1016/j.heliyon.2023.e19447

The following are the Supplementary data related to this article:

Video S1 Video S1

The following are the Supplementary data related to this article:

Video S2 Video S2

The following are the Supplementary data related to this article:

Video S3 Video S3

## 4 Conclusion

The system implemented will simulate capacitive touch-based MCC movement using ESP32. Mouse actions were implemented, including moving upward, downward, left, right, and left- and right-click. Additional functions, such as scrolling, window enlargement and reduction, window closing, etc. can be performed as a market-available mouse performs. Under a simple operating system, this method was developed. This technology enables the creation of a wide variety of applications with the minimum number of resources possible, using various application programs.

## Author contribution statement

Myda Arif; Performed the experiments.

Muhammad Hamza Zulfiqar: Conceived and designed the experiments; Performed the experiments; Wrote the paper.

Muhammad Atif Khan: Analyzed and interpreted the data; Wrote the paper.

Muhammad Zubair: Contributed reagents, materials, analysis tools or data.

Muhammad Qasim Mehmood: Conceived and designed the experiments; Analyzed and interpreted the data; Wrote the paper.

Yehia Massoud: Contributed reagents, materials, analysis tools or data.

## Data availability statement

Data will be made available on request.

## Declaration of competing interest

The authors declare that they have no known competing financial interests or personal relationships that could have appeared to influence the work reported in this paper.
