# Supplementary material for: Paper-based facile capacitive touch arrays for wireless mouse cursor control pad
Source: Heliyon. 2023 Aug 26;9(9):e19447. doi: 10.1016/j.heliyon.2023.e19447 (PMC10481284; doi:10.1016/j.heliyon.2023.e19447)
Supplement: Multimedia component 1 [file mmc1.docx]

# Supplementary Information

# Paper-Based Facile Capacitive Touch Arrays for Wireless Mouse Cursor Control Pad

# Myda Arif^1,$^, Muhammad Hamza Zulfiqar^2,$^, Muhammad Atif Khan^3^, Muhammad Zubair^3,*^, Muhammad Qasim Mehmood^1,*^, Yehia Massoud^3,*^

^1^MicroNano Lab, Department of Electrical Engineering, Information Technology University (ITU) of the Punjab, Ferozepur Road, Lahore 54600, Pakistan

^2^Department of Biomedical Engineering, Narowal Campus, University of Engineering and Technology (UET), Lahore 54890, Pakistan

^3^Innovative Technologies Laboratories (ITL), King Abdullah University of Science and Technology (KAUST), Saudi Arabia

* Correspondence: [yehia.massoud@kaust.edu.sa](mailto:yehia.massoud@kaust.edu.sa), qasim.mehmood@itu.edu.pk, muhammad.zubair.3@kaust.edu.sa

**^$^** Contributed equally

#
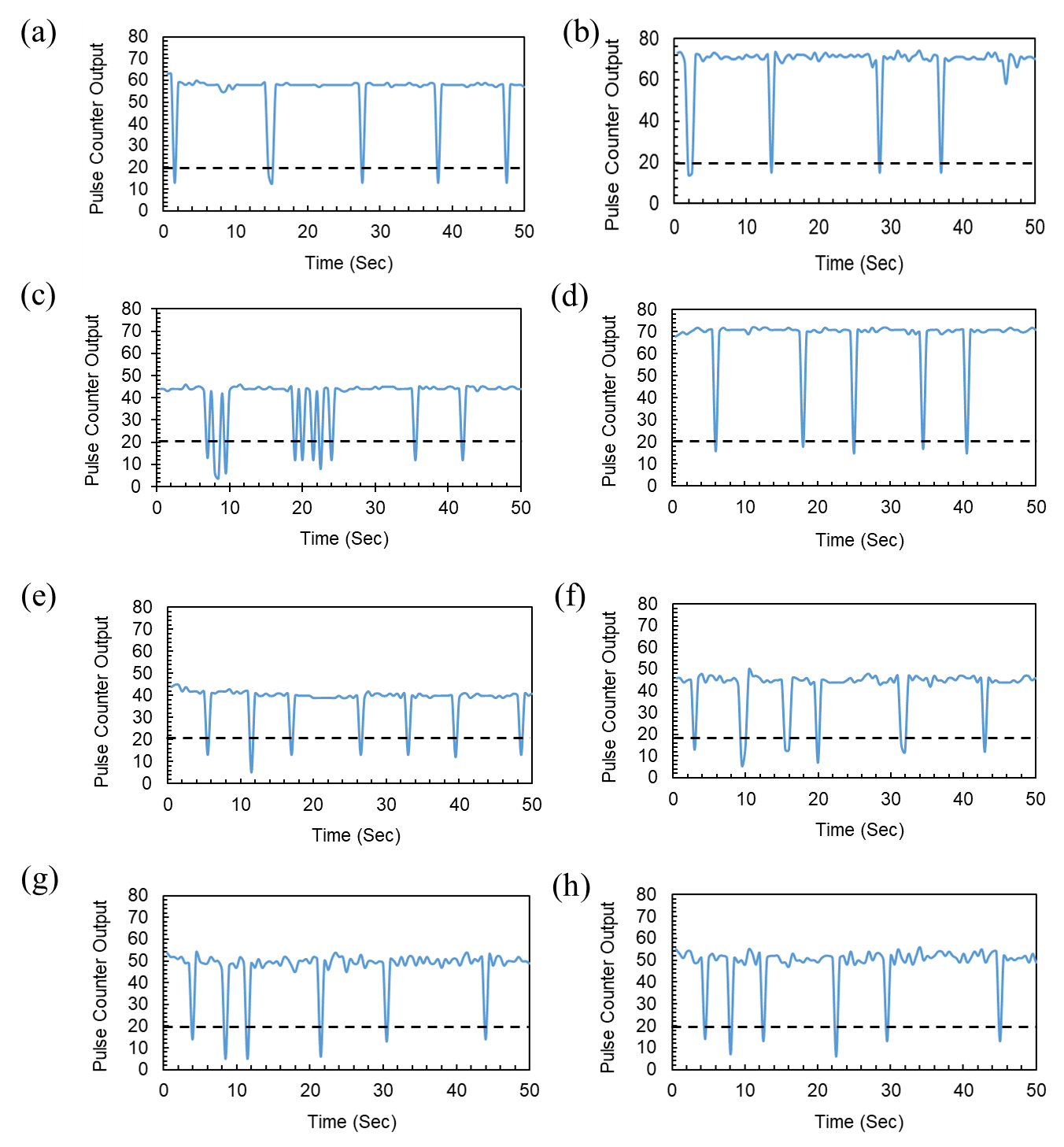


# Figure S1. Touch sensor pulse counter output of individual horizontal strips, and vertical strips arrays, left and right clicks during touched and not touched state with a bare finger. (a) Left-click. (b) Right-click. (c) Row 1. (d) Row 2. (e) Row 3. (f) Column 1. (g) Column 2. (h) Column 3.

#
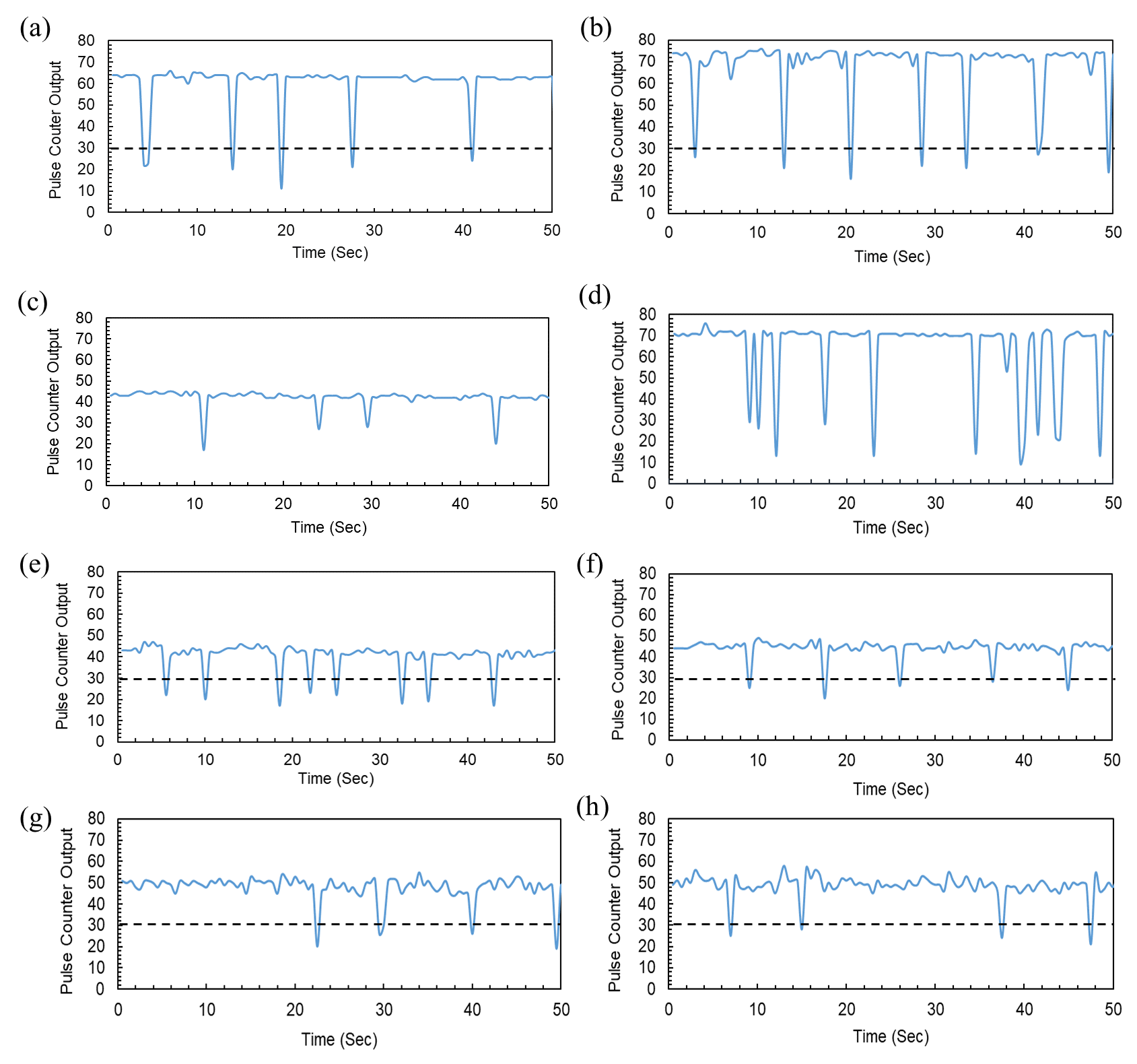


# Figure S2. Touch sensor pulse counter output of individual horizontal strips and vertical strips arrays left and right clicks with a polythene glove during touched and not touched state with a polythene gloved finger. (a) Left-click. (b) Right-click. (c) Row 1. (d) Row 2. (e) Row 3. (f) Column 1. (g) Column 2. (h) Column 3.

#
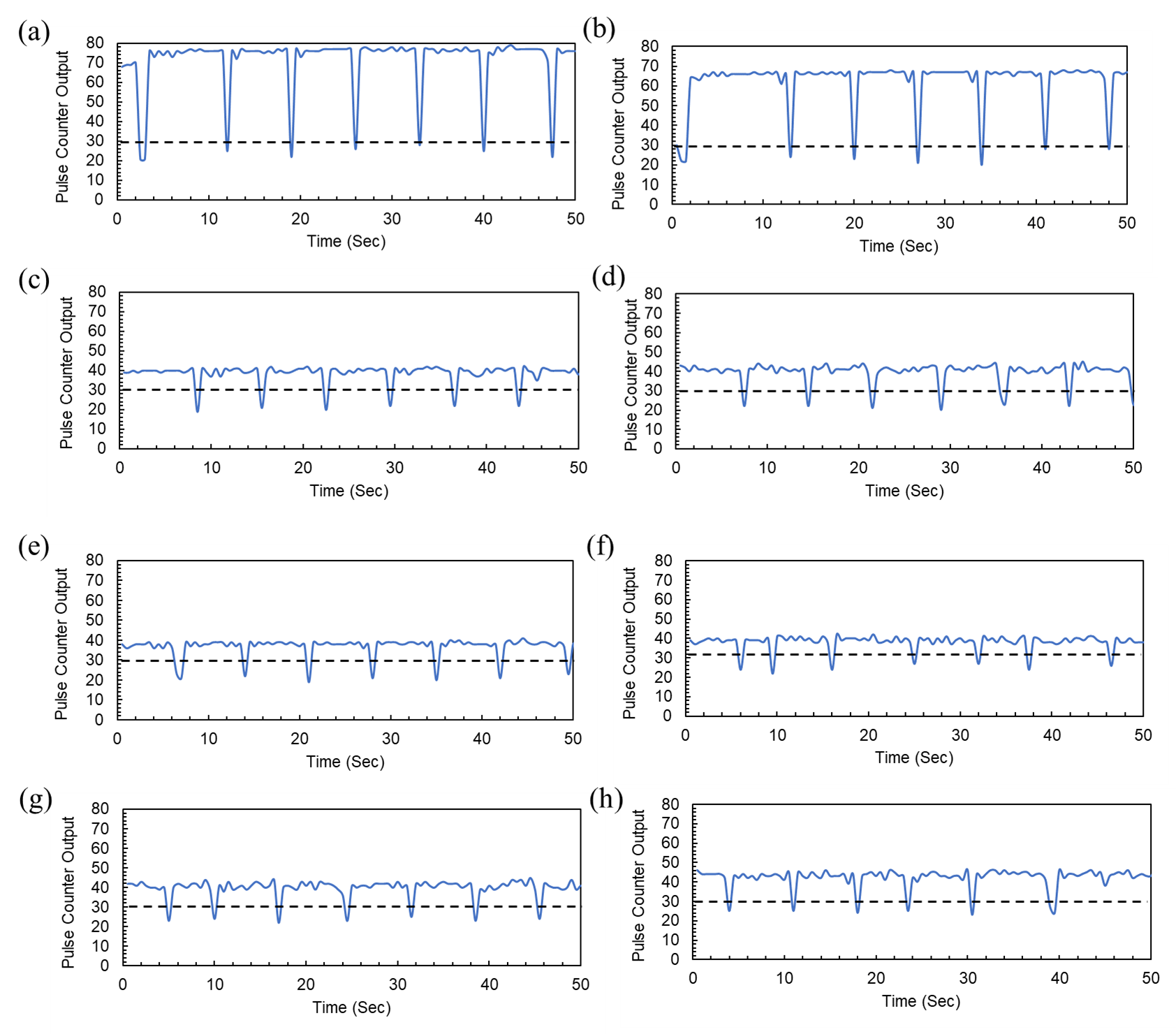


# Figure S3. Touch sensor pulse counter output of individual horizontal strips and vertical strips arrays left and right clicks with a polythene glove during touched and not touched state with a nitrile gloved finger. (a) Left-click. (b) Right-click. (c) Row 1. (d) Row 2. (e) Row 3. (f) Column 1. (g) Column 2. (h) Column 3.

# The realistic application of turning a touchpad into a flexible and wearable touchpad on the arm is shown in Figure S4.

#
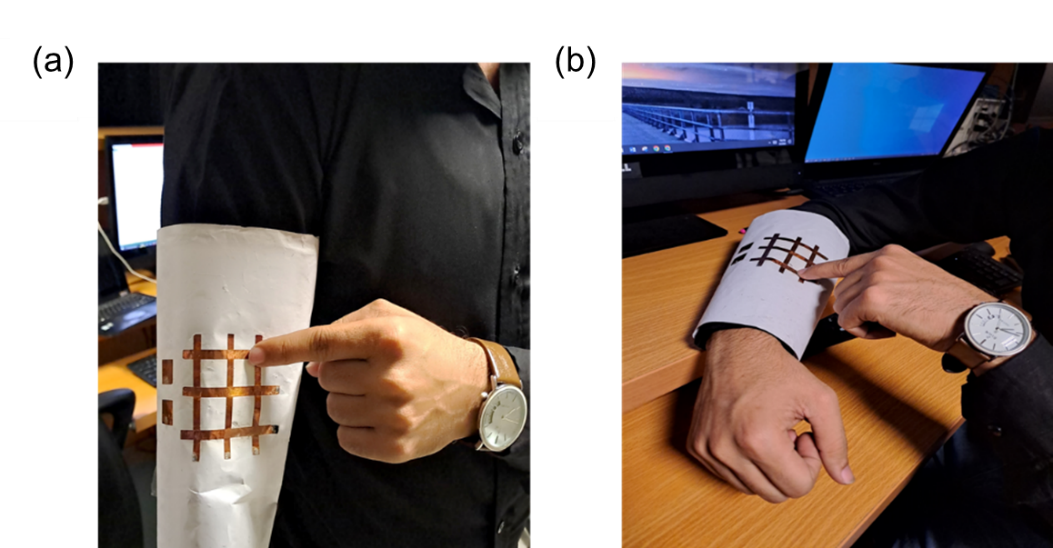


**Figure S4.** The wireless capacitive touchpad on (a) the upper arm and (b) the lower arm.
